# Hypoxia-Responsive Mesoporous Nanoparticles for Doxorubicin Delivery

**DOI:** 10.3390/polym10040390

**Published:** 2018-03-30

**Authors:** Shakera Khatoon, Hwa Seung Han, Jueun Jeon, N. Vijayakameswara Rao, Dae-Woong Jeong, M. Ikram, T. Yasin, Gi-Ra Yi, Jae Hyung Park

**Affiliations:** 1 School of Chemical Engineering, Sungkyunkwan University, Suwon 16419, Korea; shakerarizvi@gmail.com (S.K.); snaseung@gmail.com (H.S.H.); wndmsdl3451@gmail.com (J.J.); vijaychem2004@rediffmail.com (N.V.R.); daeng2ya@gmail.com (D.-W.J.); yigira@gmail.com (G.-R.Y.); 2 Department of Physics and Applied Mathematics, Pakistan Institute of Engineering and Applied Sciences (PIEAS), Islamabad 45650, Pakistan; masroor@pieas.edu.pk; 3 Department of Material Engineering, Pakistan Institute of Engineering and Applied Sciences (PIEAS), Islamabad 45650, Pakistan; yasintariq@yahoo.com; 4 Biomedical Institute for Convergence at SKKU (BICS), Sungkyunkwan University, Suwon 16419, Korea

**Keywords:** hypoxia, mesoporous silica nanoparticles, nitroimidazole, β-cyclodextrin, doxorubicin

## Abstract

Hypoxia, or low oxygen tension, is a common feature of solid tumors. Here, we report hypoxia-responsive mesoporous silica nanoparticles (HR-MSNs) with a 4-nitroimidazole-β-cyclodextrin (NI-CD) complex that is acting as the hypoxia-responsive gatekeeper. When these CD-HR-MSNs encountered a hypoxic environment, the nitroimidazole (NI) gatekeeper portion of CD-HR-MSNs disintegrated through bioreduction of the hydrophobic NI state to the hydrophilic NI state. Under hypoxic conditions, the release rate of doxorubicin (DOX) from DOX-loaded CD-HR-MSNs (DOX-CD-HR-MSNs) increased along with the disintegration of the gatekeeper. Conversely, DOX release was retarded under normoxic conditions. In vitro experiments confirmed that DOX-CD-HR-MSNs exhibit higher toxicity to hypoxic cells when compared to normoxic cells. Confocal microscopy images indicated that DOX-CD-HR-MSNs effectively release DOX into SCC-7 cells under hypoxic conditions. These results demonstrate that CD-HR-MSNs can release drugs in a hypoxia-responsive manner, and thus are promising drug carriers for hypoxia-targeted cancer therapy.

## 1. Introduction

Hypoxia, which is a pathological condition of low partial pressure of oxygen, is a common feature of solid tumors and various intractable diseases, such as rheumatoid arthritis, cardiopathy, and ischemia [1,2,3,4]. Hypoxic areas play a role in disease progression by remodeling the extracellular matrix and reforming the overall biochemical conditions around the celsls [5]. Hypoxia also plays an important role in invasiveness, metastasis, and resistance to radiotherapy and chemotherapy. Tumor tissue has regions with gradients of oxygen tension because of abnormal tumor vasculature and the resultant sluggish blood flow [6,7,8]. Oxygen concentration decreases from the surface to the interior of tumor tissue and may reach very low levels (<5 mmHg), which are much lower than the oxygen concentration in normal tissue (~70 mmHg) [4]. Owing to its typical distribution in the tumor microenvironment, hypoxia has been considered to be a novel marker for tumor-specific drug delivery via reduced oxygen partial pressure [9,10,11].

Most approaches to hypoxia-responsive drug delivery have utilized nitroaromatic or quinone derivatives due to up-regulation of bioreductive enzymes, including nitroreductase, azoreductase, and quinone reductase in many human cancers [7,12,13]. Of the derivatives that have investigated to date, nitroimidazoles (NIs) have been most widely used in biomolecule development for cancer imaging and therapy due to their high sensitivity to hypoxic environments. In such environments, hydrophobic NIs are converted to hydrophilic NIs by a series of bioreductions via a six-electron transfer process and NI can be covalently bound to cellular macromolecules in hypoxic tissues [14,15]. Given both the superior enzymatic activity of NI in hypoxia and its potential for covalent bonding, in this report, we introduced NI molecules to mesoporous nanoparticles for efficient hypoxia-targeted drug delivery.

Mesoporous silica nanoparticles (MSNs) are attractive as nanocarriers for cancer therapy and imaging due to their highly effective stimuli-responsive on-off gatekeeping capability, excellent biocompatibility, facile surface functionalization, and high pore volume for drug loading [16,17,18,19,20]. Through the introduction of gatekeepers at the surface pore, MSNs allow for the zero-release of guest molecules and site-specific delivery of entrapped drugs in response to physicochemical stimuli, such as pH, redox, biological molecules, and temperature in the tumor-microenvironment [21,22,23,24,25].

Herein, we report doxorubicin-loaded hypoxia-responsive MSNs (DOX-CD-HR-MSNs) with a nitroimidazole-cyclodextrin (NI-CD) complex as the hypoxia-sensitive gatekeeper for tumor-hypoxia targeted drug delivery. The NI of the complex can act in the hypoxic tumor-microenvironment as a trigger to release anticancer drugs from the mesopores of DOX-CD-HR-MSNs through the dissociation of the complex via bioreduction of the NI stalk. We investigated the hypoxia-sensitivity and the corresponding drug release behavior of DOX-CD-HR-MSN under hypoxic conditions using in vitro physicochemical analyses and cell experiments and confirmed the possibility of using these nanoparticles in hypoxia-responsive drug delivery.

## 2. Materials and Methods

### 2.1. Materials

Tetraethyl orthosilicate (TEOS, product number = 131903), cetyltrimethylammonium bromide (CTAB, product number = H5882), 3-aminopropyltrimethoxysilane (3-APTES), 1-ethyl-3(3-dimethylaminopropyl)carbodiimide (EDC), *N*-hydroxysuccinimide (NHS), 4-nitroimidazole (NI), doxorubicin.hydrochloride (DOX.HCl), tetrabutylammonium iodide (TBAI), poly(ethylene glycol) (PEG, MW = 10 kDa), and potassium carbonate (K_2_CO_3_) were purchased from Sigma-Aldrich (St. Louis, MO, USA). α-methyl bromoacetate was purchased from Alfa Aesar (Ward Hill, MA, USA). AquaMaxUltra water purification system (Younglin Co., Anyang, Korea) was used for deionized water that was used in the experiments. Squamous cell carcinoma (SCC)-7 cells from the American Type Culture Collection (Rockville, MD, USA) were used for in vitro cell experiments. All of the reagents were used as received without any further purification.

### 2.2. Preparation of MSN and CD-HR-MSNs

MSNs were synthesized by slight modification of procedure, as described previously [26,27]. CTAB (0.2 g) was dissolved in 100 mL of deionized water (DIW) with stirring at 25 °C for 2 h. After that, 0.7 mL of NaOH (2 M) solution was added to the CTAB solution and the resulting mixture was kept in an oil bath at 80 °C for 2 h. Then, 1.34 mL of TEOS was added in the solution with stirring for 4 h. Unreacted TEOS was removed by four cycles of extensive washing with ethanol and centrifugation at 3000 rpm for 5 min. The product obtained was dried in a vacuum oven at 80 °C for 8 h, and CTAB was removed by calcination at 540 °C for 6 h.

To prepare CD-HR-MSN, MSNs were chemically modified using the previously reported. First, the amino group-functionalized MSNs (NH_2_-MSNs) were prepared by reacting MSNs (100 mg) with 100 µL of 3-APTES in 10 mL of anhydrous toluene at 80 °C for 24 h. The NH_2_-MSNs were then purified by sequential centrifuging at 13,000 rpm for 10 min with excess toluene, methanol, and DIW. The product obtained was dried under vacuum at 45 °C for 24 h. 

Second, to accord excellent solubility to MSN, carboxylated PEG (PEG-COOH) was prepared by reported literature [28]. Briefly, Dried PEG (3 g) that was dissolved in 1,4-dioxane (8 mL) was mixed with succinic anhydride (5 mmol), 4-(dimethylamino) pyridine (DMAP) (5 mmol), and trimethylamine (TEA) (5 mmol) and stirred for 24 h at room temperature. The resulting solution was evaporated and dissolved in chloroform for filtration. After that, the filtrate was precipitated with cold diethyl ether, and filtered and dried in vacuum oven for 12 h. 

Third, to examine hypoxia responsivity, a carboxylated NI (NI-COOH) was prepared, as previously reported [29]. In brief, NI (1.00 g, 8.89 mmol), TBAI (0.075 g, 0.20 mmol), potassium carbonate (1.80 g, 13.0 mmol), and freshly distilled acetonitrile (10 mL) were mixed at room temperature under nitrogen for 30 min. Methyl bromoacetate (0.84 mL, 9.14 mmol) was added dropwise, and the system was heated at reflux for an additional 40 min. The liquid reagents were filtered off and the solid product washed with acetonitrile. The crude solid was recrystallized in ethyl acetate to yield a white NI-COO. Hydrolysis of NI-COO was carried out by refluxing with water for 24 h. The product was filtered off and placed under vacuum for 24 h to obtain NI-COOH. 

Thereafter, the NI-COOH and PEG-COOH reacted with the NH_2_-MSNs in the presence of EDC and NHS. Briefly, NI-COOH (0.3 g, 1.4 mmol) and PEG-COOH (0.1 g, 10 nmol) were dissolved in DMF and mixed with EDC (1.0 g, 5.4 mmol) and NHS (0.6 g, 5.4 mmol). The solution was mixed with the NH_2_-MSNs (200 mg) and was stirred for 24 h. The resulting solution was washed with DMF, methanol, and DIW to remove unreacted species. The product was dried under vacuum for 24 h to obtain hypoxia-responsive MSNs (HR-MSNs).

Fourth, HR-MSNs were converted to CD-HR-MSNs by reacting with β-CD. HR-MSNs (30 mg) that were suspended in 10 mL of DIW were mixed with β-CD (80 mg). The solution was stirred at room temperature overnight and washed with DMF, methanol, and DIW. The product was dried in a vacuum oven at 45 °C for 24 h to obtain HR-MSNs with β-CD gatekeepers (CD-HR-MSNs).

### 2.3. Preparation of DOX-Loaded CD-HR-MSNs (DOX-CD-HR-MSNs)

To prepare DOX-CD-HR-MSNs, HR-MSNs (50 mg) were mixed with DOX.HCl (5 mg) in 10 mL of DIW and stirred at room temperature for 24 h in the dark. Thereafter, β-CD (120 mg) in 1 mL of DIW was added to the solution and was stirred for 12 h. The resulting solution was centrifuged at 13,000 rpm for 10 min with DIW until no more DOX appeared in the supernatant. The supernatants that were collected were used to calculate the amount of unloaded DOX by measuring the absorbance at 480 nm using a UV/VIS spectrophotometer (Optizen 3220UV, Mecasys Co., Ltd., Daejeon, Korea). The product was dried in a vacuum oven at 45 °C to obtain a red powder of DOX-CD-HR-MSNs. The loading efficiency and content of DOX were calculated using the following formulas:

Loading content (%) = ((DOX in feed − amount of DOX in supernatant)/weight of DOX loaded nanoparticles)) × 100

Loading efficiency (%) = ((DOX in feed − amount of DOX in supernatant)/DOX in feed)) × 100.

### 2.4. Characterization of CD-HR-MSNs

The chemical structures of NI derivatives were confirmed by ^1^H-NMR (500 MHz, Varian Unity INOVA, Palo Alto, CA, USA). The size distribution, polydispersity index (PDI) and the surface charge of each sample were measured by a Zetasizer Nano ZS90 (Malvern Instruments, Worcestershire, UK). The morphologies of MSN and CD-HR-MSNs were observed using an HR-TEM (JEOL-2100F, Tokyo, Japan) that was operated at an accelerating voltage of 200 kV. For HR-TEM, the samples were prepared using a copper grid coated with carbon film 200 mesh (CF200-CU, Electron Microscopy Sciences, Hatfield, PA, USA). The characterizations of functionalized MSNs were carried out by using Fourier transform infrared (FT-IR) (Bruker IFS-66/S, Bruker Co., Billerica, MA, USA). The total surface area and the average pore diameter were evaluated by N_2_ adsorption/desorption measurement (Tristar II 3020, Micromeritics, Norcross, GA, USA). The surface areas and pore diameters were measured by the Brunaur-Emmett-Teller (BET) and Barrett-Joyner-Halenda (BJH) methods, respectively. The X-ray diffraction (XRD) patterns were investigated with an X-ray diffractometer using Cu-K radiation (D8 ADVANCE, Bruker Co., Billerica, MA, USA). The hypoxic state was maintained using a CO_2_/O_2_ incubator (Vision Scientific Co. Ltd., Seoul, Korea).

### 2.5. In Vitro DOX Release from DOX-CD-HR-MSNs

To investigate the DOX release profiles in hypoxic and normoxic states, DOX-CD-HR-MSNs were dispersed in PBS (pH 7.4) and the solution was transferred to cellulose membrane tubes (MWCO = 1 kDa). The dialysis tube was immersed in degassed PBS with 100 µM NADPH. Dissolved gases in media were continuously removed by degassing nitrogen gas for entire period of the release experiment to maintain hypoxic conditions [30,31]. For the control experiment, the sample was immersed in PBS containing 100 µM NADPH without degassing. Each sample was gently shaken at 37 °C and 100 rpm. The medium was refreshed at predetermined time intervals, and the DOX concentration was determined by measuring absorbance at 480 nm using a UV/Vis spectrophotometer.

### 2.6. In Vitro Cytotoxicity and Cellular Uptake of DOX-CD-HR-MSNs

SCC-7 cells were seeded at a density of 1 × 10^4^ cells/well in 96-well flat-bottomed plates in 200 μL of RPMI 1640 medium (Gibco, Grand Island, NY, USA) with 10% FBS and 1% antibiotic-antimycotic solution (100 X). After 24 h of growth at 37 °C in a humidified 5% CO_2_ atmosphere, the cells were incubated with 200 μL of fresh medium containing serial dilutions of CD-HR-MSNs. After 24 h, 20 μL of 3-(4,5-dimethylthiazol-2-yl)-2,5-diphenyltetrazolium bromide solution (5 mg/mL in PBS) was treated with each well, and the cells were additionally incubated for 4 h at 37 °C. Thereafter, the medium was removed, and the cells were dissolved in DMSO. The absorbance at 570 nm was measured using a microplate reader (BioTek, Seoul, Korea). The half maximal inhibitory concentration (IC_50_) was calculated by regression analysis of cell viability [32,33].

To observe the cellular uptake of DOX-CD-HR-MSNs, SCC-7 cells (1 × 10^5^ cells/well) were seeded onto gelatin-coated coverslips on 6-well plates and were incubated for 24 h. The cells were then washed twice with DPBS, after which the DOX-CD-HR-MSN-treated cells were incubated under hypoxic or normoxic conditions for 6 h. The cells were then washed three times with PBS and fixed with 4% formaldehyde solution. For nuclear staining, the cells were incubated with 4,6-diamino-2-phenylindole (DAPI) for 10 min. Intracellular localization of DOX was observed using an LSM 510 META NLO confocal laser scanning microscope (Carl Zeiss Microimaging GmbH, Jena, Germany).

## 3. Results and Discussion

### 3.1. Synthesis of DOX-CD-HR-MSNs

**Figure 1:**
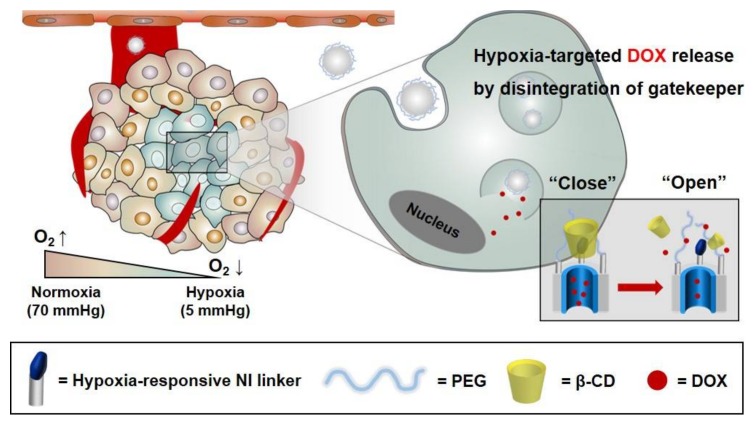
Schematic representation of doxorubicin-loaded hypoxia-responsive mesoporous silica nanoparticles with a β-CD gatekeeper (DOX-CD-HR-MSNs) for DOX delivery.

MSNs offer a biocompatible multifunctional platform with an intrinsically high surface area and porosity that are capable of delivering chemotherapeutic agents and antibiotics. In particular, MSNs readily accommodate stimulus-responsive functionalization to enable the on-command release of drug cargo in response to a variety of stimuli. In this study, for hypoxia-specific drug release in the tumor microenvironment, CD-HR-MSNs were prepared by introducing a hypoxia-responsive gatekeeper, composed of the hypoxia-responsive NI stalk and β-CD. This gatekeeper can act in hypoxic tumors as an effective trigger to release anticancer drugs from the mesopores of MSNs (Figure 1).

**Figure 2:**
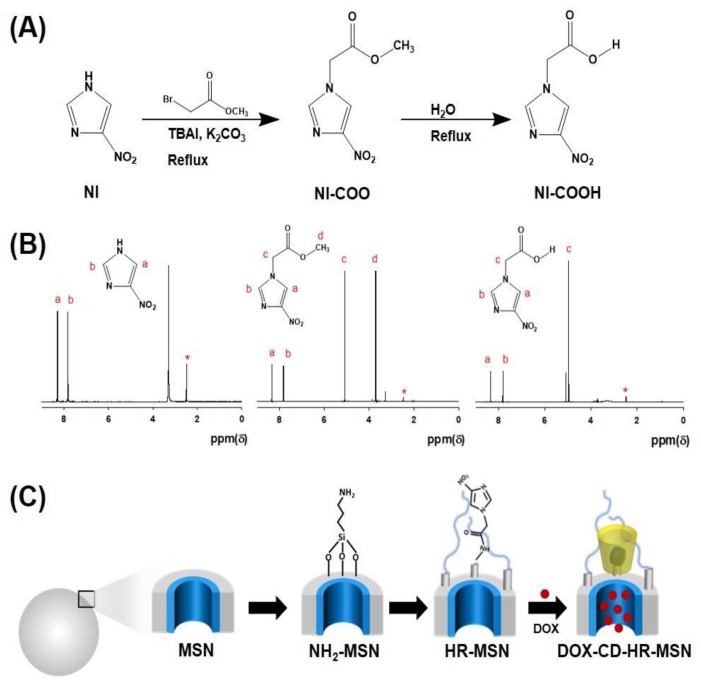
(**A**) Synthetic scheme of nitroimidazole (NI) derivatives; (**B**) ^1^H-NMR spectra of NI derivatives in DMSO-*D*_6_; (**C**) Synthetic illustration for formation of DOX-CD-HR-MSNs.

The synthetic steps for the preparation of hypoxia-responsive NI-derivatives are shown in Figure 2A. In the first step, NI was reacted with methyl bromoacetate to give the corresponding imidazole acetate (NI-COO). Next, the NI-COO was hydrolyzed to obtain the corresponding NI-COOH. Chemical structures of NI derivatives were confirmed by ^1^H-NMR (Figure 2B). The predominant characteristic peaks at δ 4.96 (s, 2H), 7.80 (s, 1H), 8.33 (s, 1H), and 3.78 (s, 3H) indicate the formation of intermediate NI-COO. Furthermore, the hydrolysis of NI-COO was confirmed by the disappearance of the signal at δ 3.78 (s, 3H), which showed the successful formation of NI-COOH.

DOX-CD-HR-MSNs were prepared following a three-step process as shown in Figure 2C. First, an amine group was introduced onto the surface of the MSN via a post-synthetic grafting method using 3-APTES to obtain NH_2_-MSN. Second, a hypoxia-responsive NI stalk and hydrophilic PEG were conjugated with NH_2_-MSN through the formation of an amide bond via simple EDC/NHS coupling in order to obtain the well-dispersed hypoxia-responsive MSN (HR-MSN) in aqueous condition. Third, DOX-CD-HR-MSN was prepared by sequential addition of DOX and β-CD. In this study, β-CD was chosen as a gatekeeper because of its unique binding nature with various organic molecules [34].

After physical encapsulation of DOX into HR-MSNs, β-CD was added to obtain DOX-CD-HR-MSNs. The loading efficacies of DOX for DOX-MSNs and DOX-CD-HR-MSNs were 82.1% and 62.5%, respectively. The corresponding DOX contents were 16.4% and 17.8%, respectively. Similarly, other researchers have demonstrated that MSNs can effectively encapsulate DOX [35,36,37].

### 3.2. Characterization of CD-HR-MSNs

**Figure 3:**
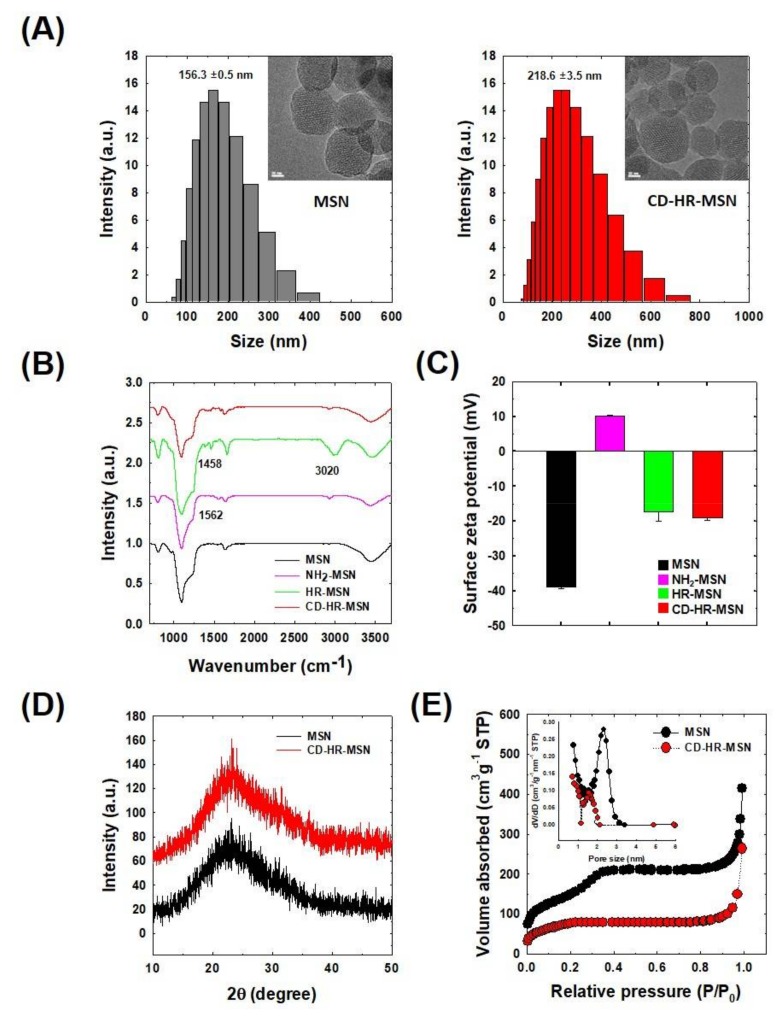
Physicochemical characteristics of functionalized MSNs. (**A**) TEM images and size distributions; (**B**) FT-IR spectra; (**C**) Change of surface zeta potential; (**D**) X-ray diffraction (XRD) spectra; and, (**E**) N_2_ adsorption/desorption isotherms.

TEM images revealed that the MSNs and CD-HR-MSNs exhibited uniform spherical natures with a hexagonal array of mesopore channels, serving as reservoirs for drug encapsulation (Figure 3A). After chemical modification of MSNs, CD-HR-MSNs did not show any destruction of their porous framework. DLS measurements demonstrated that MSN and CD-HR-MSN were well-dispersed homogeneity in the aqueous condition and had a unimodal size distribution with an average diameter of 156 nm (PDI: 0.105) and 218 nm (PDI: 0.17), respectively. The increase in size upon the modification of MSN was attributed to anchoring the PEG molecule and bulky NI-CD complex onto the surface. The chemical structures of MSNs, NH_2_-MSNs, HR-MSNs, and CD-HR-MSNs were confirmed by their FT-IR spectra (Figure 3B). The MSNs exhibited strong absorption peaks at 1097 cm^−1^ and 800 cm^−1^, providing evidence of the network structure of SiO_2_. The peak at 3458 cm^−1^ is correspondingly assigned to –OH groups that are present on the surface of MSNs. The structure of NH_2_-MSNs was confirmed by the presence of a peak at 1562 cm^−1^, which relates to the N-H bending vibration. Prominent in HR-MSNs, the PEG peak for C-H stretching at 3020 cm^−1^ and the imidazole NO_2_ symmetric stretch at 1458 cm^−1^ suggest the presence of the PEG and imidazole NI stalk on the surface of the nanoparticles. The structure of CD-HR-MSNs displayed a reduced intensity at 1458 cm^−1^, indicating the inclusion of the imidazole group in the cavity of β-CD.

Surface modification of the CD-HR-MSNs was also confirmed by measuring the zeta potential of nanoparticles (Figure 3C). MSN showed a zeta potential of −38.9 ± 1.1 mV, due to the presence of negatively charged silanol groups [26,35]. The zeta potential of NH_2_-MSN was increased to +10.2 ± 0.2 mV, implying the successful introduction of positively charged amino groups on the surface of MSNs. When NH_2_-MSNs were reacted with PEG and the NI stalk, the zeta potential of HR-MSNs was reduced to −17.3 ± 0.3 mV, indicating the capping of terminal amino groups of NH_2_-MSNs with carboxylic groups of the NI stalk and PEG. After the capping of HR-MSNs with β-CD, the zeta potential of CD-HR-MSNs showed a surface potential of −18.9 ± 0.3 mV. The wide-angle XRD spectra of the MSNs and CD-HR-MSNs exhibited a single broad peak (2θ = 20–30) that was attributed to the amorphous mesoporous silica matrix (Figure 3D). The pore characteristics of MSNs and CD-HR-MSNs were investigated by N_2_ adsorption/desorption isotherm (Figure 3E). The surface area of the MSNs and CD-HR-MSNs were 505 m^2^/g and 359 m^2^/g, respectively. The average pore size of the MSNs and CD-HR-MSNs were 2.3 nm and 1.5 nm, respectively. The decrease in both parameters indicated that the mesopores were successfully closed by the gatekeeper of the NI stalk with ß-CD grafted onto the surface of the MSNs to prevent the premature release of the drug under normal physiological conditions. The weight percent of ß-CDs on the surface of CD-HR-MSN was calculated to 3.5 wt % using glucose assay kit.

**Figure 4:**
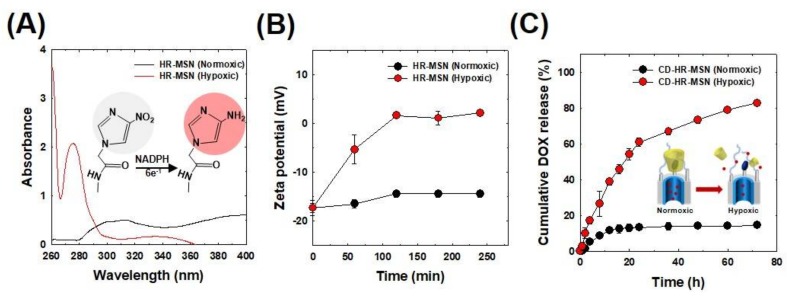
(**A**) UV/VIS spectrum of hypoxia-responsive mesoporous silica nanoparticles (HR-MSN) incubated under normoxic and hypoxic conditions for 3 h. Measurements were performed in PBS buffer containing 100 μM NADPH as an electron donor. The illustration shows the reduction of nitro to amine under hypoxic conditions; (**B**) Time-dependent change in surface charge potentials of HR-MSN incubated under normoxic and hypoxic conditions; and, (**C**) In vitro DOX release profiles from DOX-CD-HR-MSN.

The hypoxia sensitivity of the nanoparticles was evaluated by measuring the change in the absorption peaks in PBS (pH 7.4) under normoxic (20% O_2_, 5% CO_2_) or hypoxic conditions (0.1% O_2_, 5% CO_2_) for 3 h at 37 °C (Figure 4A). The characteristic NI peak appeared at 310 nm under normoxic conditions. On the other hand, under hypoxic conditions, a new peak of aminoimidazole appeared at 270 nm with the disappearance of the NI peak at 310 nm. This behavior indicated that the nitro group (NO_2_) of NI was converted to an amino group (NH_2_) under a low oxygen environment [38] via the reduction of nitro to amine via the six electron transfer process [15]. The structural change of nanoparticles in normoxic or hypoxic conditions was also assessed by measuring their zeta potentials (Figure 4B). No significant change occurred in the zeta potential of HR-MSNs under normoxic conditions for 4 h. Interestingly, under hypoxic conditions, the zeta potential value of HR-MSNs changed from −17.3 to 2.1 mV. This increase in surface charge was attributed to the presence of positively charged amino groups generated via the reduction of the nitro group of the NI stalk on the surface of HR-MSNs.

### 3.3. In Vitro Hypoxia-Responsive DOX Release Behavior of DOX-CD-HR-MSNs

To evaluate tumor hypoxia-responsive drug release behavior, DOX-CD-HR-MSNs were placed in degassed PBS buffer containing 100 µM NADPH. The amount of DOX released from DOX-CD-HR-MSNs was quantitatively measured with a UV/VIS spectrophotometer (Figure 4C). Under normoxic conditions, 10% of the DOX was released from the nanoparticles in three days, suggesting that drug release was effectively inhibited by the NI-CD complex as the bulky gatekeeper. Conversely, under hypoxic conditions, 84% of the DOX was released from DOX-CD-HR-MSNs after three days. Overall, these results demonstrated that the NI-CD complex was a robust diffusion barrier to DOX in normoxic conditions. Moreover, the hydrophobic nitro groups of NI-CD complex under hypoxic conditions were effectively bioreduced by electrons from NADPH and were altered to the hydrophilic amino group, promoting the dissociation of gatekeepers and the subsequent rapid release of DOX.

### 3.4. In Vitro Cytotoxicity and Intracellular DOX Release

**Figure 5:**
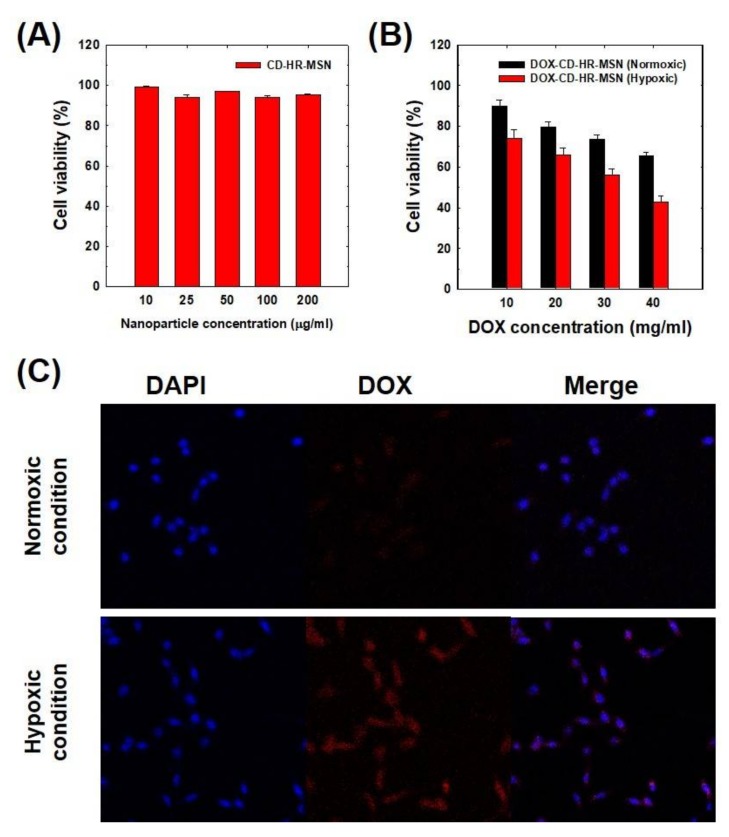
(**A**) In vitro cytotoxicity of CD-HR-MSNs; (**B**) Dose-dependent cytotoxicity of DOX-CD-HR-MSNs; and, (**C**) In vitro confocal microscopic images of SCC-7 cells incubated with DOX-CD-HR-MSNs under normoxic and hypoxic conditions.

The cytotoxicity of CD-HR-MSNs toward SCC-7 cells was measured using the MTT assay. As shown in Figure 5A, CD-HR-MSNs did not induce significant cytotoxicity up to 200 μg/mL, implying their excellent biocompatibility. Figure 5B shows cytotoxicities of DOX-CD-HR-MSNs under normoxic or hypoxic conditions. Significantly, DOX-CD-HR-MSN under hypoxic conditions led to stronger cytotoxicity when compared to that of DOX-CD-HR-MSN under normoxic conditions. Particularly, the IC_50_ value of DOX-CD-HR-MSNs under hypoxic conditions (33.7 μg/mL) was lower than that of DOX-CD-HR-MSN under normoxic conditions (60.3 μg/mL). This result might be attributed to the enhanced DOX release in response to a hypoxic environment.

To investigate the intracellular distribution of DOX, DOX-CD-HR-MSN-treated cells were incubated in normoxic (20% O_2_, 5% CO_2_) or hypoxic conditions (0.1% O_2_, 5% CO_2_) and the fluorescence signal was observed using confocal microscopy (Figure 5C). DOX-CD-HR-MSNs under normoxic conditions showed a weak fluorescence signal because of the slow DOX release by closed pores of DOX-CD-HR-MSNs. However, in the case of DOX-CD-HR-MSNs under hypoxic conditions, strong fluorescence signal of DOX was observed. This should be attributed to the fast release of DOX from DOX-CD-HR-MSNs by the disintegration of the gatekeeper under hypoxic conditions. These results are in agreement with the in vitro release behavior. Therefore, it is evident that the hypoxia-responsive DOX-CD-HR-MSNs are a unique silica-based system that are capable of effective and site-specific delivery of drugs in the tumor-microenvironment.

## 4. Conclusions

In this study, CD-HR-MSNs with a NI-CD complex as the hypoxia-responsive gatekeeper were prepared for tumor hypoxia-targeted drug delivery. The NI-CD gatekeeper on the surface of CD-HR-MSNs effectively obstructed the release of the encapsulated DOX via diffusion barrier. In hypoxic conditions with NADPH, the DOX-CD-HR-MSNs showed rapid DOX release by the disintegration of the gatekeeper complex cleaved by bioreduction of the hydrophobic NI to the hydrophilic NI. In vitro cell experiments showed that the DOX-CD-HR-MSNs effectively release DOX in response to hypoxic environments, resulting in higher toxicity when compared to that of DOX-CD-HR-MSNs in normoxic conditions. Therefore, these results suggest that DOX-CD-HR-MSNs are a promising tumor-hypoxia targeted drug carrier and further imply that the nitroimidazole linker may be utilized for the development of hypoxia-responsive nanoparticles.
